# Computational analysis of polymorphic residues in maltose and maltotriose transporters of a wild *Saccharomyces cerevisiae* strain

**DOI:** 10.1515/biol-2025-1080

**Published:** 2025-04-16

**Authors:** Oscar A. Faz-Cortez, Alma Y. Sánchez-López, César I. Hernández-Vásquez, Andre Segura-Ruiz, Benito Pereyra-Alférez, Jorge H. García-García

**Affiliations:** Facultad de Ciencias Biológicas, Universidad Autónoma de Nuevo León, Instituto de Biotecnología, Nuevo León, Mexico

**Keywords:** *Saccharomyces cerevisiae*, α-glucoside transporters, polymorphisms

## Abstract

The metabolism of maltose and maltotriose, the primary sugars in brewing wort, depends on an efficient transport system. However, most *Saccharomyces cerevisiae* strains transport maltotriose inefficiently, leaving residual α-glucosides in the final product. Proteins involved in maltotriose transport exhibit diverse polymorphic sequences linked to sugar transport efficiency. In this study, a wild *S. cerevisiae* strain was placed under adaptive selection, resulting in a strain with a 65 and 44% increase in maltose and maltotriose transport rates, respectively. Genes encoding maltose and maltotriose transporters, including *MAL*x1, *MPH*x, and *AGT*1, were detected in both the native and adapted strains. One variant of Mal31p, carrying a polymorphism at position 371 in transmembrane helix 7, was identified. This helix has been reported to have a high likelihood of undergoing polymorphisms. Bioinformatics analysis revealed structural changes affecting substrate interactions and channel dynamics, with the polymorphism conferring greater protein flexibility and reducing electrostatic interactions. These results suggest that the residue at position 371 in maltose and maltotriose transporters is a key element distinct from those previously reported. Additionally, we propose a significant set of polymorphic residues within these transporters potentially resulting from the evolution of these proteins.

## Graphical abstract

**Figure:**
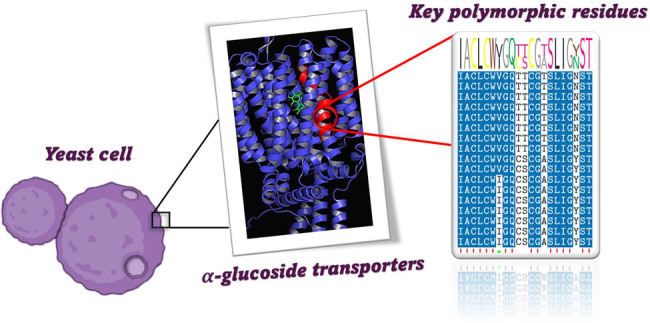

## 1 Introduction

Beer is one of the most significant biotechnological products of our time and one of the pioneering products of biotechnology. Brewer’s yeast produces ethanol from sugars present in wort in a process that implies efficient transport and metabolism of maltose and maltotriose, the most abundant sugars in a typical beer wort, representing approximately 65 and 17.5%, respectively [1].

Inefficient sugar consumption has been identified as an important issue in wort fermentation, where multiple physical and chemical factors play a significant role in their uptake, especially in yeast strains associated with *Saccharomyces cerevisiae* [2–4]. The presence of genes encoding α-glucoside transporter permeases, such as *MPH*2, *MPH*3, *AGT*1, and *MTT*1 (also known as *MTY*1), is also important, as well as the presence of five unlinked *loci* (MAL1–4 and MAL6). The canonical structure of MAL *loci* comprises three genes: the gene encoding the sugar transporter (*MAL*x1), the gene encoding a maltase enzyme (*MAL*x2), and an activator factor responsible for the transcription induction of the other two genes within the *locus* (*MAL*x3), with x representing the specific *locus* number [5–9]. It is important to mention that *MPH*2 and *MPH*3 are not part of any MAL *locus*, unlike the *AGT*1 and *MTT*1 genes. It has been reported that the MAL1 *locus*, considered the ancestral *locus*, can harbor a *MAL*11 gene, an *AGT*1 gene, an *MTT*1 gene, or even all three genes together [5,6]. All permeases encoded by these genes belong to the major facilitator superfamily (MFS), which introduces the substrate via a proton-driven symporter and consists of 12 transmembrane helices (TMHs) [10,11].

In addition to the presence of these genes, other molecular factors influencing the proper consumption of maltose and maltotriose have been described, such as the number of copies, variations in their promoter sequences, positive regulation, and key polymorphisms in the sequences of the transporter proteins [12–18]. Polymorphisms in key TMHs, such as TMH7 and TMH11, have been reported to have a high impact, suggesting that alterations in these helices could be crucial for transporter activity [12,13,17–19].

Due to the inefficient uptake of sugar in some brewing yeast, it is important to gain insights into the sequence and structure of α-glucoside transporters. For this reason, the objective of this study was to analyze and look for *MAL*x1, *AGT*1, *MPH*x, and *MTT*1-like genes in a wild *S. cerevisiae* strain (FI20) and in a descendent (FI20-G30) that was subjected to adaptive selection for improved maltose and maltotriose transport. The sequence of Mal31p of both strains showed a substitution of an isoleucine for a valine in position 371 with a predicted effect on the protein structure and substrate interaction.

Our findings remark the importance of characterizing and further investigating key polymorphisms through computational analysis, which could play a critical role in the efficient sugar transport in these permeases, as well as potentially using specific polymorphic residues as molecular markers to predict the fermentative capacity or strains with potential for adaptation to fermentative processes. Furthermore, we propose a potential set of polymorphic residues that could be important for the efficiency of the activities of these transporters.

## 2 Materials and methods

### 2.1 Yeast strains

Yeast strains were isolated from various locations in Northern Mexico from flowers and fruits following the methodology reported previously with some modifications [20]. A sugar-rich medium was employed, prepared by grinding 100 g of malt per liter and subsequently mashing it for 1 h at 65°C and adjusted to 6 °Brix with malt extract. Petri dishes were prepared with this medium, adding 20 g of agar per liter of the medium. The collected samples were added to tubes containing malt medium and incubated for 7 days at 25°C. Subsequently, the samples were removed, and serial dilutions from 10^−1^ to 10^−5^ were performed, inoculating each dilution on malt agar plates by spreading. The plates were incubated at 25°C for 5 days. Colonies with different morphologies were observed under a microscope to confirm the presence of yeasts. Once distinct colonies were identified, they were transferred to new malt agar plates for isolation. After obtaining different isolates, a screening was performed based on their fermentative capacity and the sensory characteristics of the fermented product (data not shown). Based on these criteria, strain FI20 was selected, which was isolated from a flower from *Mammillaria carretii* in Icamole, Nuevo León, Mexico, in 2019.

Strain FI20 then underwent an adaptive selection process through serial cultivation in wort with increasing sugar concentration prepared with 100–350 g of malt per litter and adjusted with malt extract. The yeast was incubated in 50 mL tubes containing 10 mL of wort with a sugar concentration starting at 6 °Brix and reaching up to 21 °Brix in increments of 0.5 °Brix. The culture was incubated under anaerobic conditions at 25°C for 7 days, and after each fermentation cycle, the yeast was inoculated in wort with the next higher concentration of sugar. After 30 cycles, strain FI20-G30 was obtained.

### 2.2 Yeast identification by PCR-RFLP and ITS-5.8S sequencing

FI20 and FI20-G30 strains were identified by PCR-RFLP and by 5.8S rRNA gene, ITS1 and ITS2 sequence, using the ITS1 and ITS4 primers [21] (Table S1). Amplifications were performed in a 100 µL volume in a Veriti 96-well thermal cycler (Applied Biosystems). The reactions were carried out using the following program: pre-incubation (94°C for 1 min), 35 amplification cycles (94°C for 30 s, 60°C for 30 s, 72°C for 30 s), and a final extension cycle (72°C for 5 min). Subsequently, 5 µL of the reaction products were stained with GelGreen and observed on 1.5% agarose gels. Two *S. cerevisiae* strains were used as controls: the S288C strain and the US-05 (Fermentis Lille, France). PCR products were purified and subject to RFLP analysis using endonuclease *Hae*III, and DNA nucleotide sequence. The restriction products were observed on 3% agarose gels and dyed with GelGreen. While nucleotide sequence was determined with Sanger sequencing, the nucleotide sequences were analyzed using BLAST (https://blast.ncbi.nlm.nih.gov).

### 2.3 Cellular transport rate of maltose and maltotriose

We conducted the transport assay as previously described [22] in our *S. cerevisiae* strains FI20, FI20-G30, and S288C. To estimate the transport of maltose and maltotriose, we used *p*-nitrophenyl-α-d-glucopyranoside (pNP-glucose) and *p*-nitrophenyl-α-d-maltoside (pNP-maltose) as substrates, structurally related to maltose and maltotriose, respectively. Cells of the tested strains (15 g/L) were suspended in 50 mM succinate-Tris buffer at pH 5.0 and maintained for 5 min at 30°C. Subsequently, pNP-glucose or pNP-maltose (40 nM) was added, and 100 μL aliquots were taken over a 5-min period at 1-min intervals. Each aliquot was immediately placed in a boiling water bath for 3 min. After cooling the aliquots to room temperature, 100 μL of 2 M NaHCO_3_ was added, and the cells were centrifuged to collect the *p*-nitrophenol present in the supernatant, which was then measured at 400 nm. The transport rate was calculated using the slope of the linear uptake of each substrate over the reaction period and normalized to 1 mg of dry yeast weight. All assays were performed in triplicate, with boiled cells used as a control. The Student’s *t*-test was performed using R version 4.2.3.

### 2.4 Detection of α-glucoside transporter genes

The detection of transporter genes *MAL*x1, *AGT*1, and *MPH*x in FI20 and FI20-G30 was performed using PCR on genomic DNA. Additionally, based on the recently reported transporter ScMalt#5p in *S. cerevisiae* [17], we decided to search for this or similar genes in our strains. We selected primers for the *MTT*1 (also called *MTY*1) gene due to the high identity between this permease and ScMalt#5p.

Genomic DNA was obtained as mentioned elsewhere [23] and adjusted to a concentration of 50 ng/µL with a Nanodrop. All primers used were obtained from a previous work [24] (Table S1). The reactions were carried out in a total volume of 20 µL. A *Saccharomyces pastorianus* strain was used as a positive control for the amplification of these genes. The reactions were performed in a Veriti 96-well thermal cycler (Applied Biosystems) using the following program: pre-incubation (94°C for 2 min), 30 amplification cycles (94°C for 15 s, primer Tm for 20 s, 72°C for 30 s), and a final extension cycle (72°C for 30 s). Five microliters of the reaction products were visualized on 3% agarose gels stained with GelGreen, using a 25 bp ladder. Electrophoresis was performed at 85 V for 60 min.

### 2.5 Sequence analysis of *MTT*1 PCR products

The PCR-amplified products obtained using the *MTT*1 primers were processed similarly to the ITS-5.8S products described previously, preparing them for subsequent Sanger sequencing. Upon amplification and sequencing, the products were analyzed using BLAST (https://blast.ncbi.nlm.nih.gov) to confirm their identities. Amino acid sequences were deduced from our nucleotide sequences using the ExPASy Translate tool [25]. Topological predictions of Mal31p-FI20 and Mal31p-G30 sequences were performed using the CCTOP server [26].

To identify polymorphic regions in transmembrane helix 7 (TMH7), we carried out multiple alignment sequences using the MSA package version 1.32.0 [27], employing CLUSTAL-W with the BLOSUM80 substitution matrix. The sequences used were obtained from NCBI (https://www.ncbi.nlm.nih.gov) (Table 1).

**Table 1:** Sequences used for *in silico* analysis

Sequence name	*Saccharomyces* strain	Name in this work	Accession number
Mtt1p/Mty1p	*S. pastorianus* WS34/70	Mtt1p-1	ABV21349.1
			B8LJC8
Mtt1p/Mty1p	*S. pastorianus* A15	Mtt1p-2	ABV21348.1
Mtt1p/Mty1p	*S. pastorianus* NCYC387	Mtt1p-3	SBT28088.1
Mtt1p/Mty1p	*S. pastorianus* NCYC374-2	Mtt1p-4	SBT28087.1
Mal31p	*S. cerevisiae* S288C*	Mal31p-288	NP009857.1
			P38156
Mal31p	*S. cerevisiae* FI20*	Mal31p-FI20	PQ159167
Mal31p	*S. cerevisiae* FI20-G30*	Mal31p-G30	PQ159168
ScMalt#5p	*S. cerevisiae* W184	ScMalt#5p	LC716142.1
Mal31p	*S. cerevisiae* YJM244*	Mal31p-NB1	AJQ00677.1
Mal31p	*S. cerevisiae* YJM972*	Mal31p-NB2	AJP92160.1
Mal31p	*S. cerevisiae* YJM975*	Mal31p-NB3	AJP92548.1
Mal31p	*S. cerevisiae* YJM453*	Mal31p-NB4	AJQ04099.1
Mal31p	*S. cerevisiae* YJM1592*	Mal31p-NB5	AJP90614.1
Mal31p-1	*S. pastorianus* SpIB1	Mal31p-SpIB1	PRJNA1124045
Mal31p-5	*S. pastorianus* SpIB2	Mal31p-5-SpIB2	PRJNA1124045
Mal31p-3	*S. pastorianus* SpIB2	Mal31p-3-SpIB2	PRJNA1124045
Mal31p-7	*S. pastorianus* SpIB2	Mal31p-7-SpIB2	PRJNA1124045
Mal31p-10	*S. pastorianus* SpIB2	Mal31p-10-SpIB2	PRJNA1124045
Agt1p	*S. pastorianus* SpIB2	SpAgt1p	PRJNA1124045
Agt1p	*S. cerevisiae* INSC1006	ScAgt1p	KAF1904524.1

*Strains from non-brewing environments.

For protein structure prediction of the Mal31p-FI20/G30 polymorphism, we simulated the I371V substitution in the Mal31p-288 protein, and its 3D structure was predicted using the AlphaFold2 server [28,29], as well as that of Mal31p-3-SpIB2. The 3D structures of Mal31p-288 and Mtt1p-1 were obtained from the UniProt database (https://www.uniprot.org) (Table 1). The 3D structures of maltose (CID: 6255) and maltotriose (CID: 439586) were obtained from the PubChem database (https://pubchem.ncbi.nlm.nih.gov).

The substrate transport channels of Mal31p-288, Mal31p-FI20/G30, Mal31p-3-SpIB2, and Mtt1p-1 were predicted using the PoreWalker server [30]. Molecular docking studies were conducted using AutoDock Vina [31,32], assessing interactions between Mal31p-288, Mal31p-FI20/G30, Mal31p-3-SpIB2, and Mtt1p-1 with substrates maltose and maltotriose. All docking assays were performed with an exhaustiveness value of 8, as recommended previously [33,34]. For the predicted effect of the I371V mutation, we used the DynaMut server [35], using Mal31p-288 as the wild-type sequence. All structural visualizations were carried out using PyMOL version 3.0.3 (https://www.pymol.org).

## 3 Results and discussion

### 3.1 Molecular identification of yeasts

RFLP results for FI20 and FI20-G30 showed an identical band profile to *S. cerevisiae* strains S288C and US-05 (Figure S1), and they match the approximate sizes (320, 240, 180, and 140 bp) of the bands previously reported in *S. cerevisiae* strains [36]. Additionally, according to the ITS-5.8S DNA sequence analysis, our strains had 100% identity with the *S. cerevisiae* strain (MT136553.1). Both ITS-5.8S sequences were uploaded to the GenBank database: FI20 (PQ276518.1) and FI20-G30 (PQ276519.1).

### 3.2 Comparison in transport rate of maltose and maltotriose and molecular detection of transporter genes

The FI20 strain is a wild *S. cerevisiae* that we isolated from the environment, selected for the favorable organoleptic characteristics of its fermentation products and its more efficient growth in wort compared to the other wild strains (data not shown). This strain was subjected to high sugar concentrations to obtain a descendant strain adapted to these conditions, which we named FI20-G30. To determine whether this adaptive selection process affected the α-glucoside transport rate relevant to brewing, we conducted a comparative analysis of maltose and maltotriose transport.

**Figure 1:**
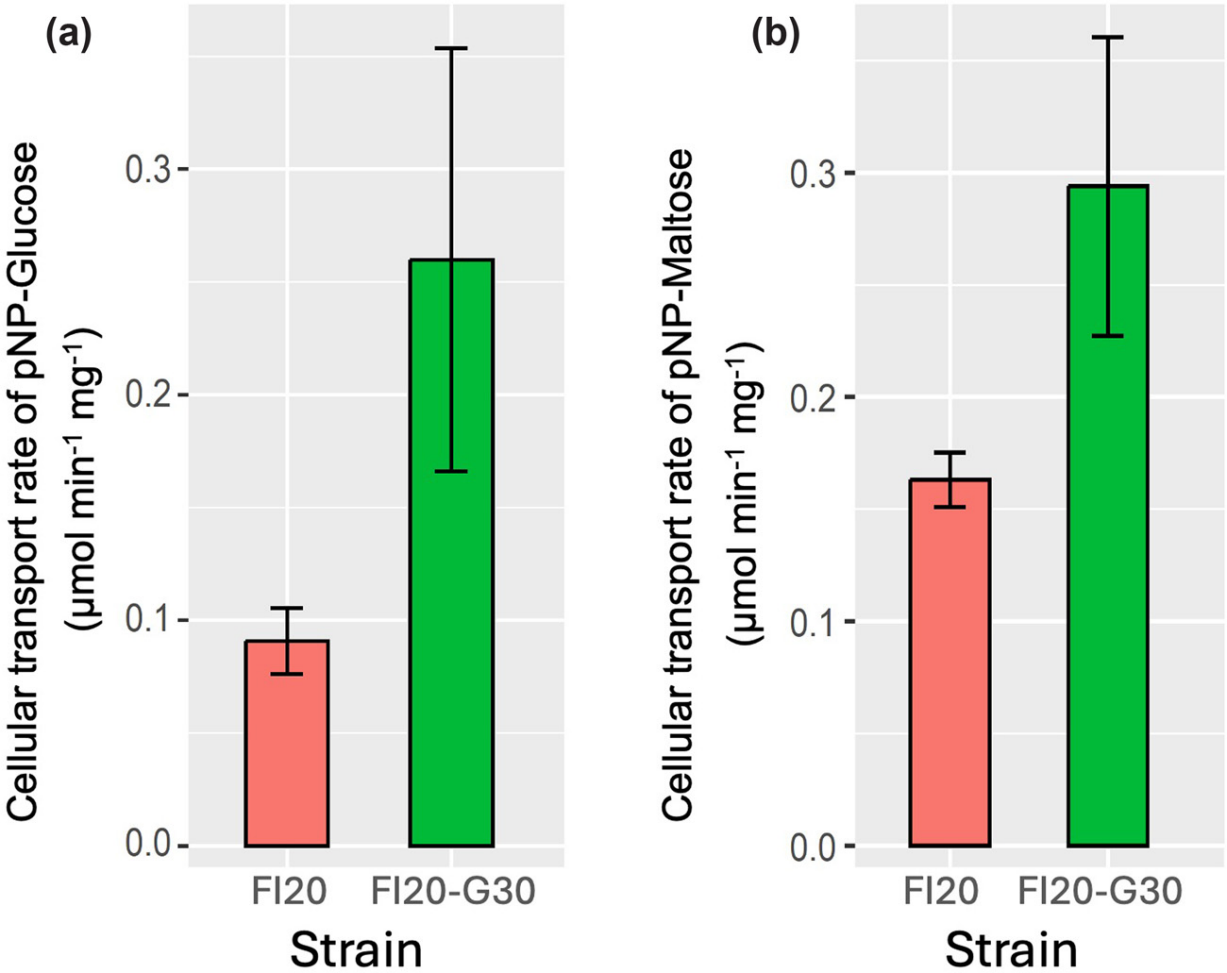
Cellular transport rate of (a) pNP-glucose and (b) pNP-maltose over 5 min in the wild-type FI20 strain and the adapted FI20-G30 strain. There is greater consumption by the FI20-G30 strain compared to FI20 in both substrates (*p* = 0.03865 and *p* = 0.04055, respectively).

The rate of pNP-glucose and pNP-maltose transport, which are related to the transport of maltose and maltotriose, respectively [18,19], revealed a significant difference between the two strains (Figure 1, Figure S2). Specifically, strain FI20 transported 0.0908 μmol min^−1^ mg^−1^ dry cell yeast of pNP-glucose and 0.1631 μmol min^−1^ mg^−1^ dry cell yeast of pNP-maltose, while strain FI20-G30 exhibited transport levels of 0.2598 μmol min^−1^ mg^−1^ dry cell yeast of pNP-glucose and 0.2938 of pNP-maltose. This corresponds to an increased transport of 65% maltose and 44% maltotriose by FI20-G30 (*p* = 0.03865) compared to FI20 (*p* = 0.04055). We observed that both strains showed higher pNP-maltose transport than pNP-glucose transport, which is not very common; however, strains exhibiting higher maltotriose transport than maltose have been reported [24].

In contrast, no transport was detected for either substrate in the laboratory strain S288C. Although the genome of this strain contains *MPH*x and *AGT*1 genes and two *MAL loci* [5,16], these genes are non-functional due to a mutation in the MAL activator [37,38]. Since the expression of *MPH*x and *AGT*1 is dependent on the MAL activator [6,7], this mutation would affect not only the expression of *MAL*x1 genes but also these other genes.

The presence of α-glucoside genes in yeasts is crucial for the brewing and baking industries [18]. Therefore, we analyzed their presence in FI20 and FI20-G30 strains by PCR amplification of the *AGT*1, *MPH*x, and *MAL*x1 genes. Additionally, we used primers for the *MTT*1 (also called *MTY*1) gene to search for it and possibly *MTT*1-like genes. This was because a transporter in an industrial brewing strain of *S. cerevisiae* with high identity to the *MTT*1 permease, named ScMalt#5p with 97% identity, was recently reported and characterized [17].

**Figure 2:**
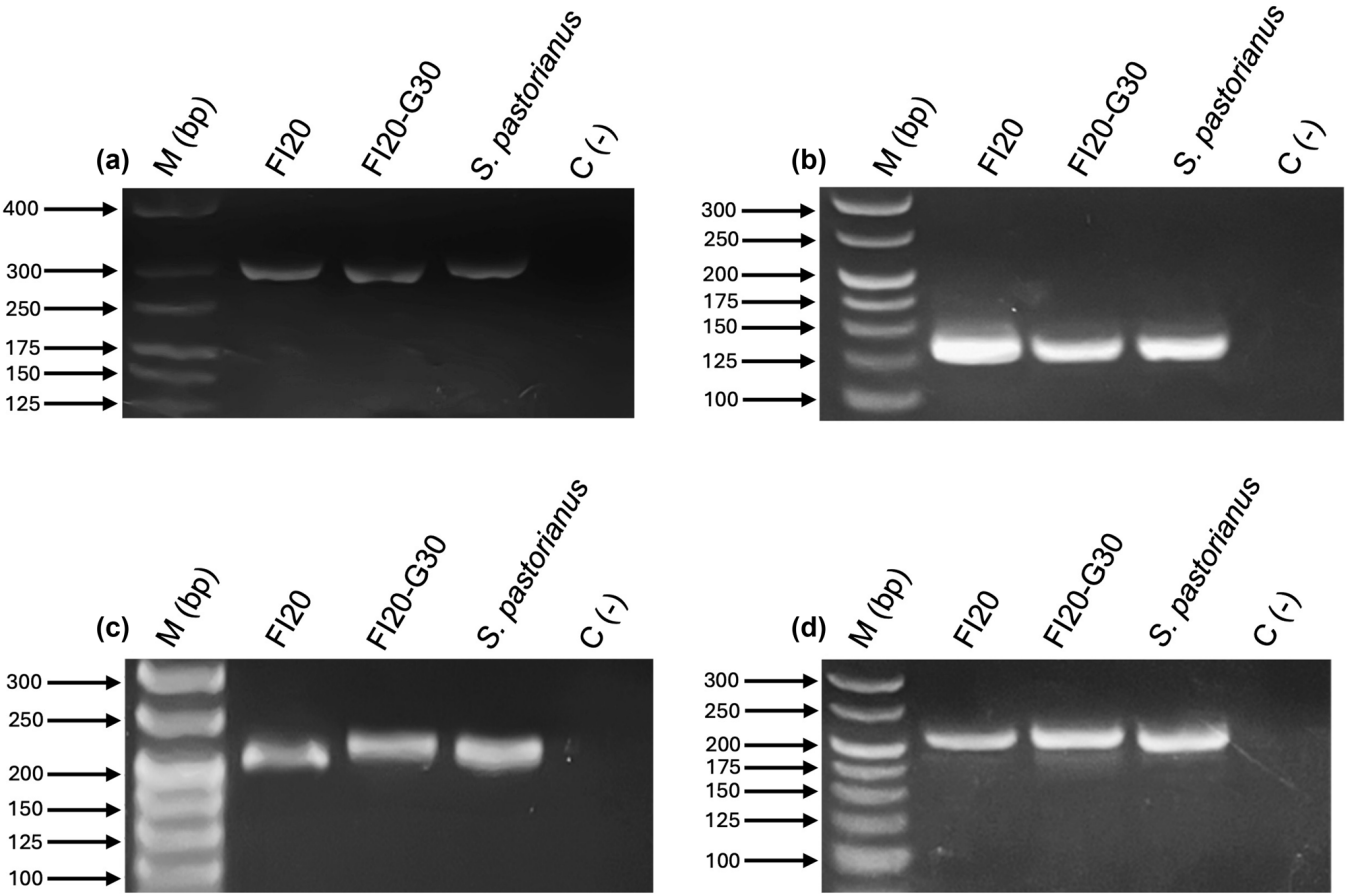
Amplification of genes encoding α-glucoside transporters in FI20 and FI20-G30 strains. Amplification of all targeted genes is observed in both strains: (a) *MAL*x1, (b) *AGT*1, (c) *MPH*x, and (d) *MTT*1. The amplicon sizes match those reported: 282 bp for *MAL*x1, 128 bp for *AGT*1, 204 bp for *MPH*x, and 201 bp for *MTT*1 [24].

We obtained amplifications with all the primers used in strains FI20 and FI20-G30. The amplicon sizes matched those reported [24]: 128 bp for *AGT*1, 282 bp for *MAL*x1, 201 bp for *MTT*1, and 204 bp for *MPH*x (Figure 2). Given the amplification in both strains using the *MTT*1 gene primers, we decided to sequence and perform bioinformatics analysis on these amplicons.

The presence of these genes in our strains provides insight into their potential fermentative capabilities [9,24]. Additionally, it is interesting that strain FI20 and its descendant, FI20-G30, possess the *AGT*1 gene, which has been reported to not only have a high affinity for maltose but also to utilize a wide range of α-glucosides, including maltotriose [6,39]. Although it has been reported that strains overexpressing *MAL*x1 (*MAL*31 and *MAL*61) are capable of transporting maltotriose [14], other authors have argued that no *MAL*x1 gene encodes a permease that transports maltotriose [6,9,17,40].

Similarly, *MPH*2 and *MPH*3 were initially characterized as maltotriose transporter genes [7]; however, other reports indicate that they are not [40,41]. The ambiguous and controversial characterization of these transporters (*MAL*x1 and *MPH*x) as maltotriose transporters has led to attributing the transport of this sugar in *S. cerevisiae* strains to the *AGT*1 gene and/or other *MTT*1-like genes such as those recently characterized in a brewing strain of *S. cerevisiae*: *ScMALT*#2 and *ScMALT*#5 [17]. Since the FI20 wild strain was isolated from a non-brewing context, the presence of the *AGT*1 gene could be responsible for the transport of pNP-maltose (related to maltotriose).

Strains not able to transport maltotriose but carrying the *AGT*1 gene can acquire the ability to transport it after an adaptive selection process due to an increase in the expression of this gene [42]. However, the presence of all genes tested provides our strains with various tools to evolve more easily in brewing environments. These genes are in subtelomeric regions, which confers even higher possibilities for genetic changes to improve transport, such as duplications, enhancements in the regulatory system, or even the generation of a new chimeric gene [41,43].

Other factors besides the presence of these genes are important for the efficient consumption of these sugars, such as the conditions in the later stages of fermentation, transporter copy number, variations in the promoter regions of these genes, and positive regulators of *Mal* transporters [14,15,18]. It has even been described that polymorphisms of a few amino acids in the TMHs of the MFS are responsible for their preference for different substrates [12,13,17–19]. Nevertheless, the approach of detecting the presence of these genes can be used for the predictive characterization of the fermentative capacity of yeasts. These results make these strains of interest, as they could continue to acquire the favorable fermentative capacity of maltose and maltotriose through constant selection processes. Additionally, we support adaptive selection as a useful and relatively simple tool for improving certain characteristics in strains with a brewing focus.

### 3.3 Sequence identification of *MTT*1 amplicons

After obtaining amplifications in the FI20 and FI20-G30 strains using the primers for the *MTT*1 gene [24], we sequenced the amplicons via Sanger sequencing and analyzed *in silico* to compare the obtained sequences with the reported transporter sequences. We translated them into amino acid sequences using the Translate tool from ExPASy [25], obtaining a 67-amino-acid sequence in both strains, with 100% identity between them, indicating no polymorphisms between the FI20 and FI20-G30 strains in the amplified region using these primers. We predicted the region of the protein obtained from our amplification using the CCTOP server [26] and identified that our amino acid sequence corresponds to 2 TMHs out of the 12 typically found in these sugar transporters [11]. In addition to the two TMHs, a cytoplasmic topological domain and an extracellular domain were also predicted.

Analyzing the sequences with BLAST (https://blast.ncbi.nlm.nih.gov), we found that the sequence has a high identity (98.51%) and 100% coverage with the *S. cerevisiae* Mal31p permease protein sequence (CBK39376.1). Aligning our sequence with that of this permease, we found that the two helices we amplified correspond to TMH7 and TMH8; however, we identified polymorphisms in the TMH7 at position 371, which was an isoleucine to valine substitution (I371V). This polymorphism is located near those reported previously in TMH7 at 378/379 and 383/384 positions in Mal61p, Mtt1p, ScMalt#2p, and ScMalt#5p. It was identified that the amino acids in these positions and others in TMH11 are crucial for determining transporter preference for maltose and/or maltotriose [17]. Additionally, other researchers have reported specific polymorphisms and key residues in TMH7 and TMH11 of maltose and maltotriose transporters in *S. pastorianus* and *Saccharomyces eubayanus* [12,18].

These findings highlight the importance of these TMHs in the transport activity of these permeases, which is why we further analyzed it through bioinformatics predictions to gain insights into the potential role this change could play in the protein structure of our strains.

### 3.4 Polymorphic regions observed in the transmembrane helix 7

To compare the TMH7 of the Mal31p sequence from our FI20 and FI20-G30 strains, we conducted a multiple sequence alignment with the TMH7 sequences from Table 1. We chose to align the sequences of Mtt1p because the primers that amplified Mal31p in our strains were initially designed to amplify *MTT*1, and ScMalt#5p was included due to its high identity with Mtt1p (97%). For these analyses, we used the amino acid positions of Mal31p to account for extra amino acids in lengths between Mal31p, Mtt1p, ScMalt#5p, and Agt1p.

**Figure 3:**
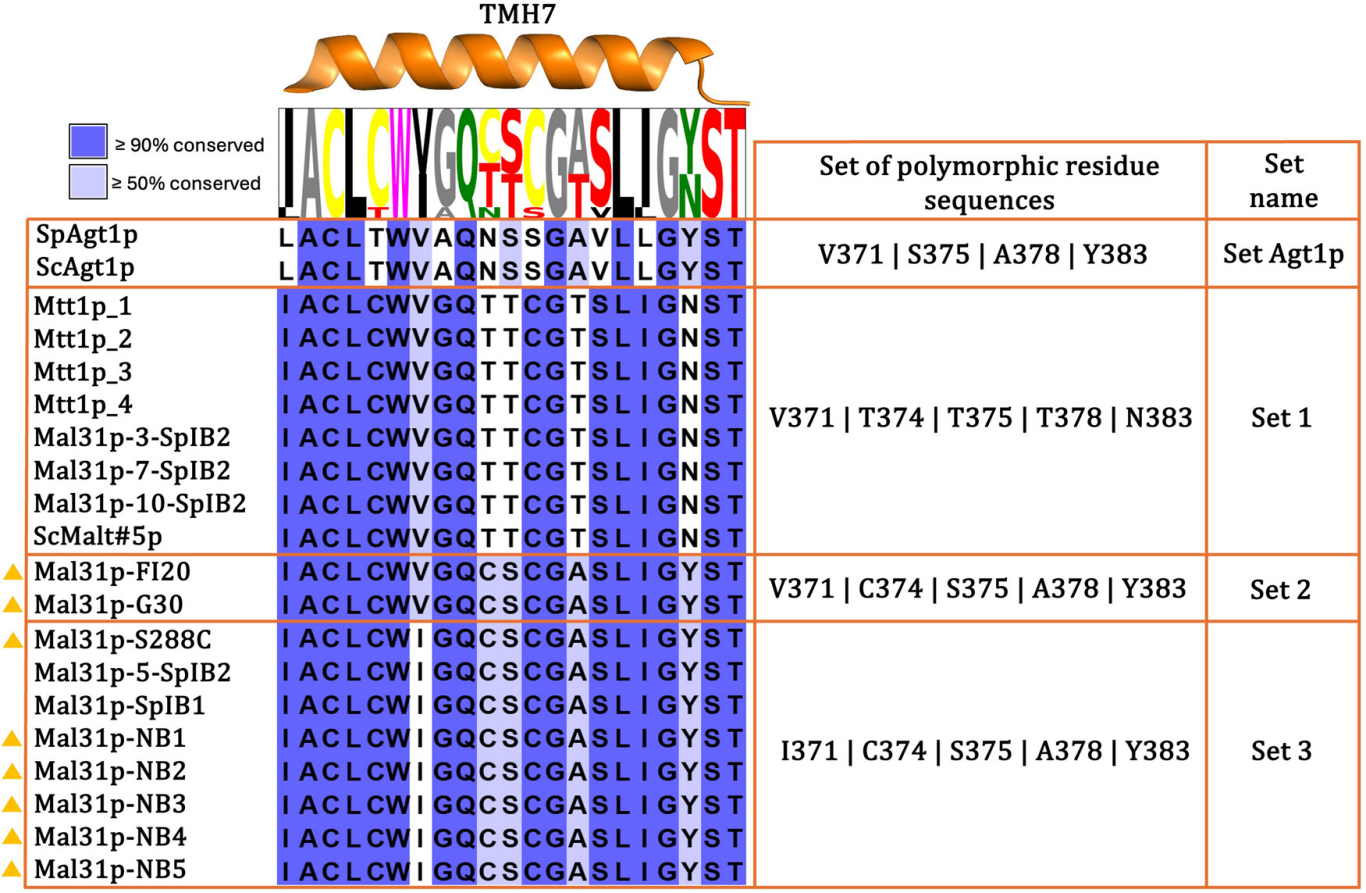
Comparison of TMH7 in Mal31p from our FI20 and FI20-G30 strains with different sequences of α-glucoside transporters (Table 1). Sequences marked with a yellow triangle are strains obtained from a non-brewing environment. Note how all sequences in set 1 come from brewing strains, while set 3 groups those from a non-brewing environment. The sequence of our strains, grouped in set 2, contains amino acids from other sets, with V371 like those in set 1 and set Agt1p and C374, S375, A378, and Y383 like those in set 3.

We found that in all the sequences of Mtt1p used, Mal31p-3-SpIB2, Mal31p-7-SpIB2, Mal31p-10-SpIB2, and ScMalt#5p (Set 1), as well as ScAgt1p and SpAgt1p (Set Agt1p), have a valine at position 371, just like Mal31p-FI20 and Mal31p-G30 (Set 2). However, the sequences grouped in set 1 do not have a high identity with our sequences in this TMH, as other polymorphisms are observed at residues 374, 375, 378, and 383. The sequences in set 1 have T374, T375, T378, and N383, while those in set 2 have C374, S375, A378, and Y383. Moreover, polymorphic residues of Mal31p-288, Mal31p-NB1-5, Mal31p-5-SpIB2, and Mal31p-SpIB1 (Set 3) are grouped, which have C374, S375, A378, and Y383, just like those in set 2, but differ at position 371, having isoleucine instead of valine (Figure 3). This means that the TMH7 of Mal31p in our strains contains amino acids from both sets (1 and 3) of sequences. On the other hand, although the Agt1p sequence differs more from the sequences grouped in sets 1, 2, and 3, the TMH7 of ScAgt1p and SpAgt1p shares some of the same residues at key positions, such as V371, S375, A378, and Y383.

It is interesting to note that only brewing strains are grouped in set 1 and set Agt1p, while strains from a non-brewing environment are grouped in sets 2 and 3, except for the brewing strains SpIB1 and SpIB2. However, the SpIB2 strain, which has three copies of Mal31p in set 1, transports more maltose (28%) and maltotriose (32%) compared to SpIB1 [18], in which no copies of Mal31p with the residues from set 1 were found. These results suggest that there might be a relationship between the polymorphic residues of set 1 and the efficiency of α-glucoside transport. It is also noteworthy that, among all sequences grouped in sets 1, 2, and 3, polymorphisms exist in these same five positions, and each residue alternates between just two amino acids (371 I or V, 374 T or C, 375 T or S, 378 T or A, and 383 N or Y).

### 3.5 Prediction of I371V mutation effects in Mal31p

The sequences of the Mal31p amplicon in our FI20 and FI20-G30 strains are identical, so from this point on, we will refer to these sequences as Mal31p-FI20/G30. Similarly, for SpMal31p-3-SpIB2, SpMal31p-7-SpIB2, and SpMal31p-10-SpIB2 from set 1, we will refer to them as SpMal31p-3-SpIB2.

**Figure 4:**
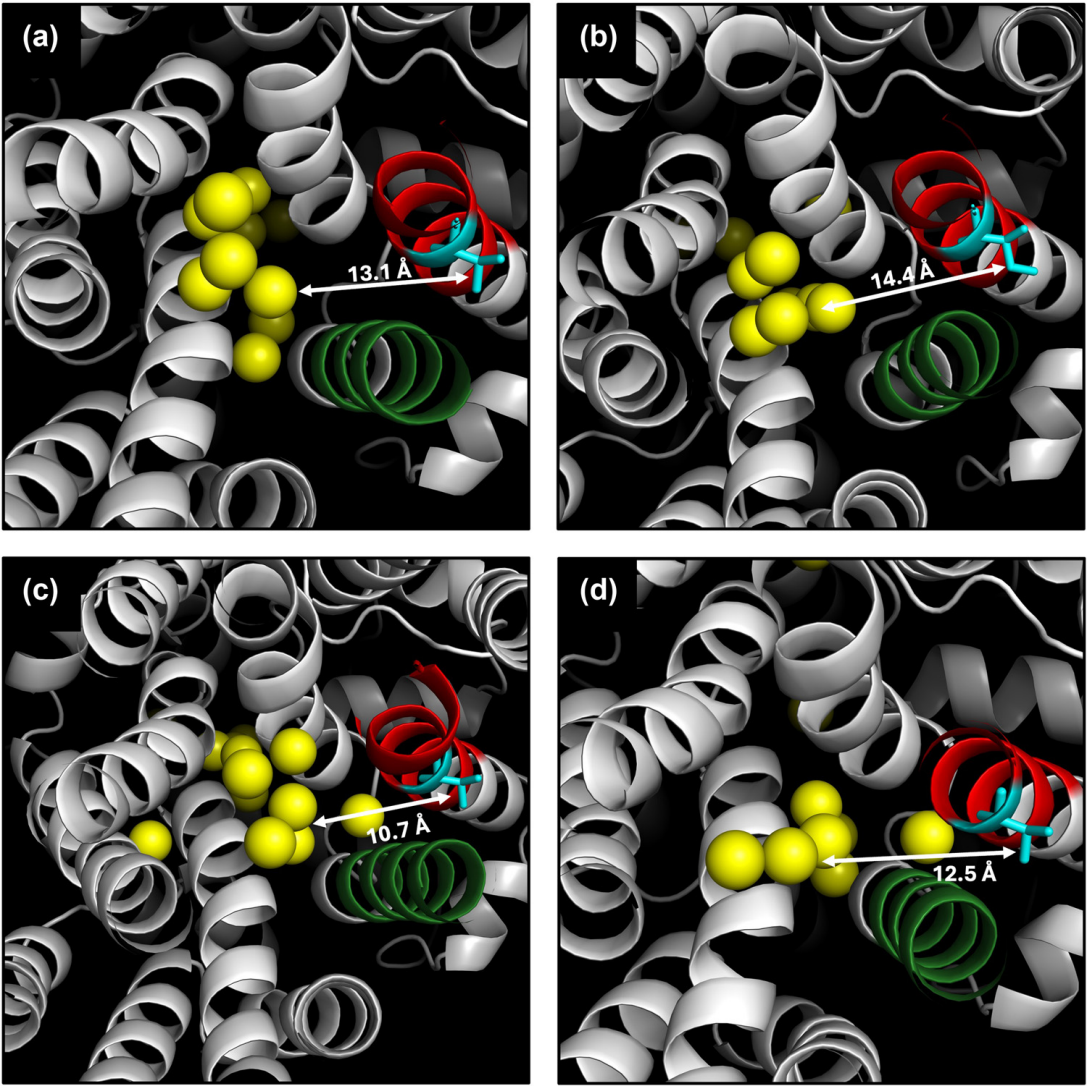
Prediction of the substrate transport channel represented as yellow spheres for Mal31p-FI20-G30 (a), Mal31p-288 (b), Mal31p-3-SpIB2 (c), and Mtt1p-1 (d). TMH7 is shown in red, TMH11 in green, and position 371 is in cyan. The distance between residue at 371 position of Mal31p-288 and the substrate transport channel is 14.4 Å, whereas, in Mal31p-FI20/G30, this distance is 13.1 Å, showing a difference between them due to the single I371V change. On the other hand, Mal31p-3-SpIB2 and Mtt1p-1 show smaller distances of 10.7 and 12.5 Å, respectively. Note that in all the transporters, the side chain of the residue at position 371 is facing TMH11.

To visualize the proximity of the residue at position 371 in the Mal31p-288, Mal31p-FI20/G30, Mtt1p-1, and Mal31p-3-SpIB2 proteins to the substrate transport channel, we conducted a prediction using the PoreWalker server [28]. We found that the helix containing the polymorphism (TMH7) is directly exposed to the substrate transport channel in all the analyzed transporters (Figure 4). The side chain of the amino acid at position 371 in the four transporters is not directly exposed toward the substrate transport channel; however, it has been discussed that despite this, such residues could indirectly influence substrate recognition by affecting other residues exposed to the substrate transport channel [17]. The side chain faces each other with TMH11, which has also been reported as important due to having key residues in α-glucoside transporters in yeasts [12,17,19,44].

According to the predictions, the I371V mutation in Mal31p would cause a change in structure, as even though it is only one amino acid substitution, there is a difference in the distance between the residue and the substrate transport channel in Mal31p-288 and Mal31p-FI20/G30. For Mal31p-288, a distance of 14.4 Å was predicted, while for Mal31p-FI20/G30, a distance was 13.1 Å. On the other hand, in Mal31p-3-SpIB2 and Mtt1p-1, smaller distances were obtained, 10.7 and 12.5 Å, respectively (Figure 4).

We used molecular docking to predict the interactions between maltose and maltotriose with the polymorphic residue groups previously identified (Figure 3) and to gain insights into the possible impact of polymorphism I371V on the transporter activity [45,46]. The transporters used for molecular docking were Mal31p-288, Mal31p-FI20/G30, Mtt1p-1, and Mal31p-3-SpIB2.

**Figure 5:**
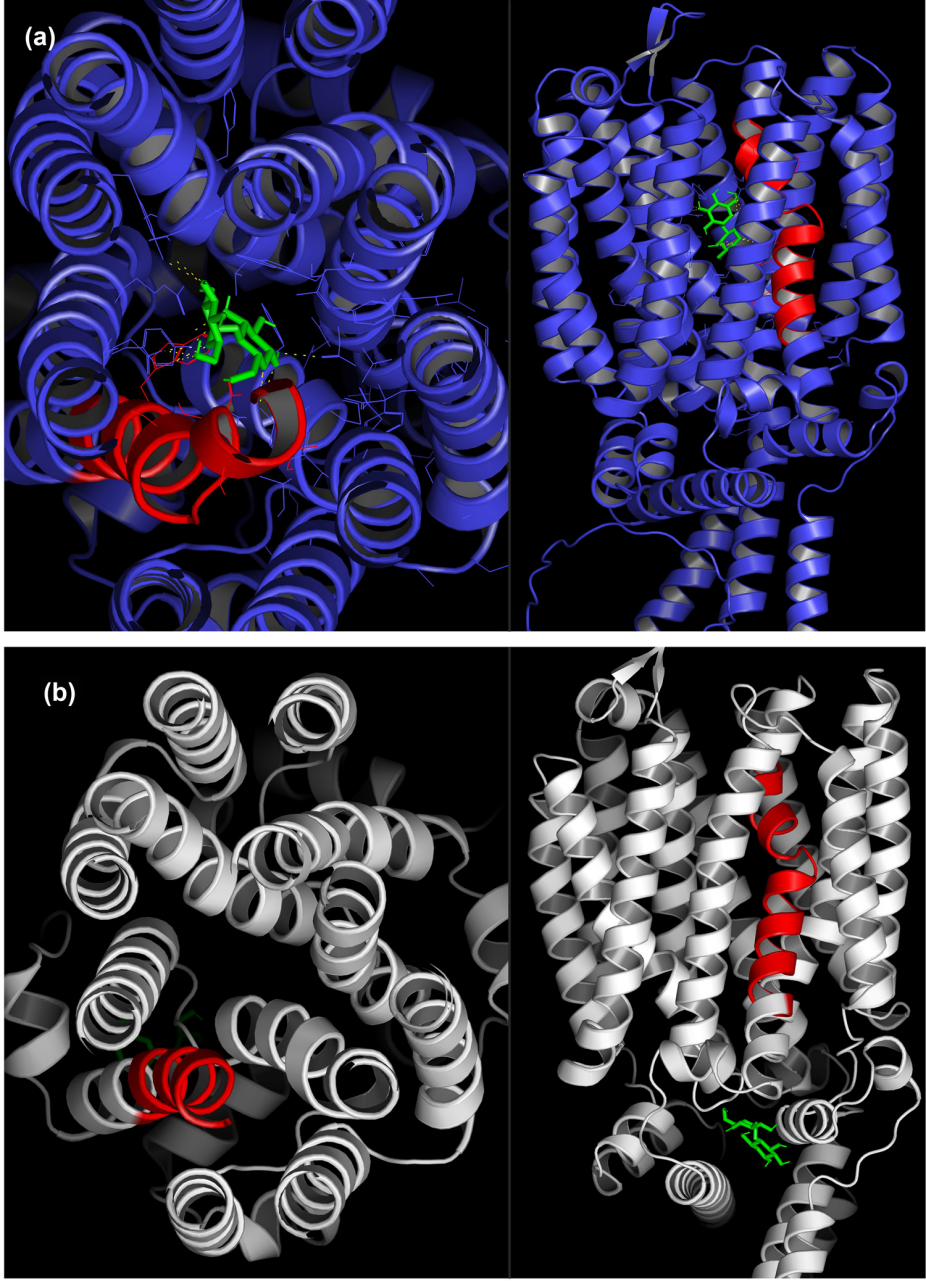
Molecular docking between the transporters Mal31p-288 (a) and Mal31p-FI20/G30 (b) with maltose. The images on the left show the protein from above, and the images on the right show a lateral view. Note the binding between the substrates and the amino acids of the substrate transport channel in the Mal31p-288 transporter, but for Mal31p-FI20/G30, no interaction between these amino acids and maltose is predicted. TMH7 is shown in red in all images, and maltose is shown in green. In the right image of Mal31p-FI20/G30, there was interaction between maltose and amino acids localized in an intracellular region, but in a real context, there would be no interaction with those amino acids unless the maltose had first entered the cell.

Docking analyses involving maltose and maltotriose with Mal31p-288 (Figure 5a), Mtt1p-1, and Mal31p-3-SpIB2 revealed interactions between the substrates and amino acids in the substrate transport channel (Figure S3), which are similar to those recently reported in a maltotriose transporter in *S. eubayanus* [12]. These results suggest that interactions with these residues would result in a stable system and energetically favorable binding, making efficient transport activity more likely [46–48]. These findings are further supported by reports that these permeases, Mtt1p-1 and Mal31p-288, are indeed functional and efficient in transporting these sugars [8,14]. On the other hand, the results of the I371V substitution would significantly alter the structure and possible activity of the protein. No binding or interaction was detected between maltose (Figure 5b) and maltotriose (Figure S3) and any amino acids in the substrate transport channel in any of the 40 different poses predicted for Mal31p-FI20/G30; instead, the simulation showed interactions in other regions different from the transport channel (Figure 5b). This suggests that this single sequence change can potentially compromise normal transport activity by failing to establish a stable interaction with the substrate transport channel [47].

To predict the effect of the I371V mutation on the stability of Mal31p, we used the sequence of Mal31p-288 as the wild-type sequence to which the mutation was introduced. The prediction was carried out using the DynaMut server [35], which predicts the vibrational entropy energy change (ΔΔS_Vib_ ENCoM) between the wild-type protein and the mutated protein. Vibration entropy is the major contributor to the configurational entropy of proteins. A negative ΔΔS_Vib_ ENCoM value represents a rigidification of the protein structure, while a positive value indicates an increase in its flexibility [49].

**Figure 6:**
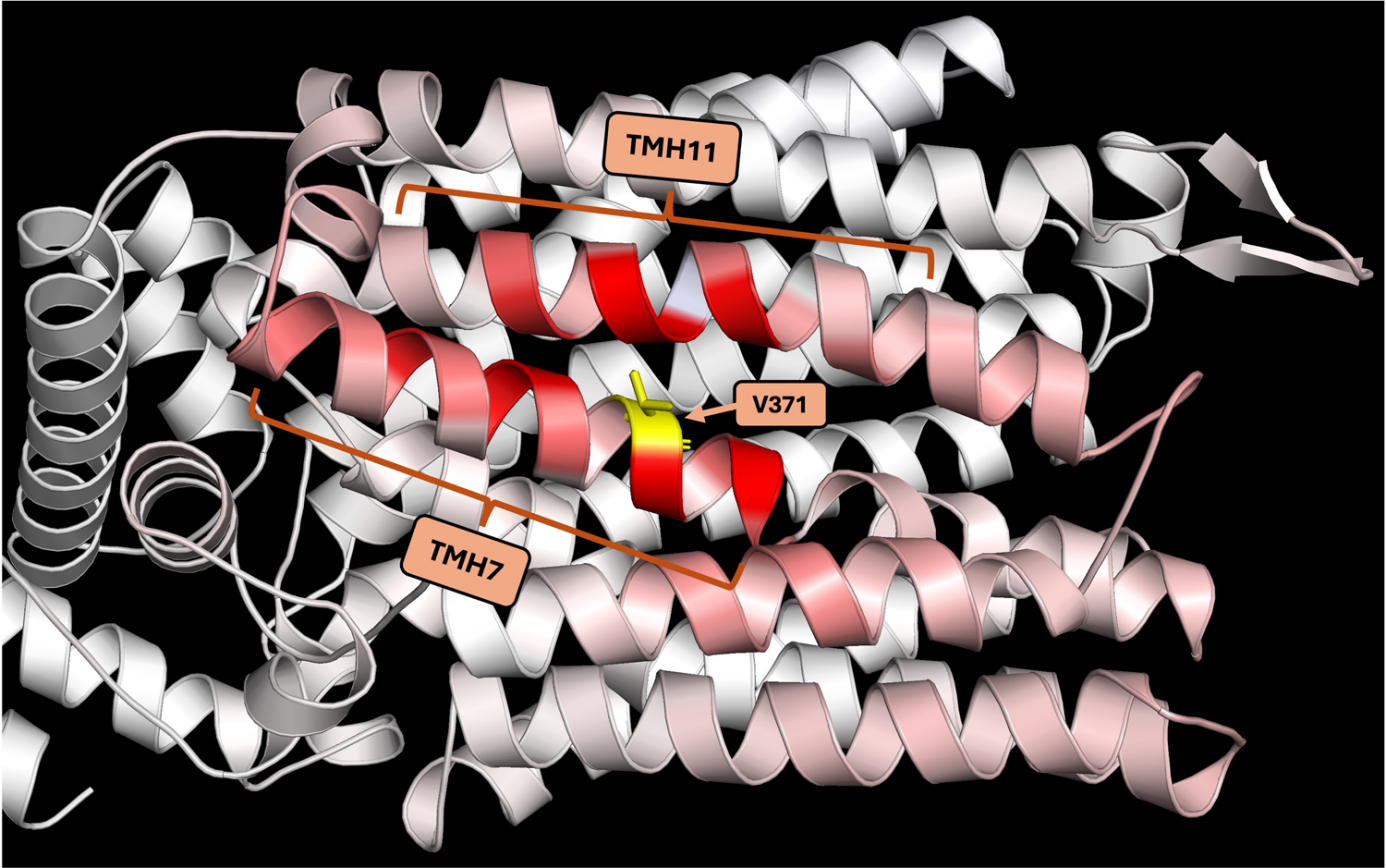
Prediction of the stability effect caused by the I371V mutation in Mal31p-288. The intensity of the red color is related to the change in ΔΔS_Vib_ ENCoM, with a higher intensity of red representing a greater change in the flexibility of the protein caused by the mutation.

According to the ΔΔS_Vib_ ENCoM, this mutation would confer greater flexibility to the protein (ΔΔS_Vib_ ENCoM: 0.136 kcal mol^−1^ K^−1^). The amino acids most affected by this mutation in terms of ΔΔS_Vib_ ENCoM are T363 (cytoplasmic topological domain); A366, C367, G372, C374, S375, C376 (of TMH7); and L496, A497, A500, Y501, V503, I504 (of TMH11) (Figure 6).

These results complement those showing that the single change at position 371 results in a difference in the distance between this residue and the substrate transport channel. Along with the molecular docking results, this suggests that the polymorphism would lead to a significant alteration in the protein. Even a single mutation can considerably impact protein function by altering rigidity or flexibility compared to the wild-type protein due to the crucial role these properties play in protein function [50,51]. In yeast, the significance of key residues for conformational flexibility in sugar transporters of the same family as those under study has been reported, with evidence indicating that a single amino acid change can have a notable effect [52].

**Figure 7:**
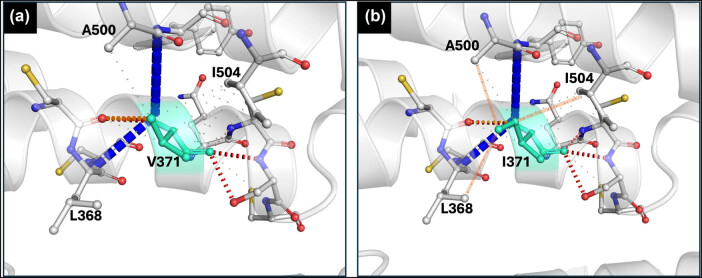
Prediction of the effect on amino acid interactions following the I371V substitution in Mal31p wild-type. Note that three interactions were lost due to the mutation (a) compared to the wild-type Mal31p (b). The amino acids where the interaction with residue 371 was lost are A500 (TMH11), I504 (TMH11), and L368 (TMH7). The interactions in the wild-type protein that were lost due to the mutation are highlighted in orange.

Additionally, the prediction of the mutation’s effect on the protein suggests that it would result in the loss of three electrostatic interactions between amino acids (Figure 7a). Specifically, the wild-type Mal31p sequence features three additional interactions compared to Mal31p-I371V (Figure 7b). The amino acids involved in these lost interactions are A500 (TMH11), I504 (TMH11), and L368 (TMH7). The loss of these intramolecular interactions is likely responsible for the increased flexibility observed in the protein structure relative to wild-type Mal31p [53]. Recent reports have established that interactions between these two TMHs in a maltotriose transporter in *S. eubayanus* are important for transport activity. They also attributed the large epistatic interaction between TMH7 and TMH11 to a single residue located in TMH7 and reported that a mutation in this key residue completely abolished transport capacity [12].

All these results support that a single polymorphism in TMH7 of MFS permeases can affect the protein, particularly if it occurs in residues around the substrate transport channel [12,17]. Our findings suggest that the residue at position 371 in these transporters is a key element, distinct from those previously reported, as we have predicted significant changes in some characteristics of the protein with this single polymorphism, which would likely impact the transporter function. However, the ability to transport maltose and maltotriose in our strains does not necessarily come from this polymorphism, as transport activity may be regulated by factors beyond its protein structure [13,15].

Additionally, we propose the set of polymorphic residues in set 1 (V371, T374, T375, T378, and N383) as a critical group of amino acids for efficient α-glucoside transport. Strains with sequences containing this set of residues in TMH7 have been previously reported to be efficient in transporting maltose, especially maltotriose. This could, in part, explain the differences in the high transport rate of SpIB2 compared to SpIB1 and our strains, which do not possess this set of residues, as well as strains from non-brewing environments [8,17,18]. While we recognize that multiple factors influence the efficient transport of these sugars, we propose that the polymorphic residues in set 1 are one of them, based on the reported importance of TMH7.

Our strains have V371 but lack T374, T375, T378, and N383, as found in the brewing yeast from set 1. We suggest that this set of polymorphisms (from set 1) might have resulted from selection over time due to the consistent exposure of these strains to high sugar concentrations in brewing environments [42,43]. This is further supported by the predicted substrate–substrate transport channel interactions from the docking analysis and the absence of such interactions in the Mal31p-FI20/G30 sequence from our wild strain.

The presence of all the analyzed transporter genes, the positive response after adaptive selection, the high transport rate of maltose and especially maltotriose analogues, and the presence of a permease that varies in TMH7 compared to previously reported permeases suggest that the FI20-G30 strain has potential to continue adapting to brewing environments. Additionally, it is interesting that it possesses the amino acid valine at position 371, which is also present in maltotriose transporters (set 1 and set Agt1p) from efficient brewing strains. According to our hypothesis that the polymorphic residues from set 1 are a result of adaptation to brewing environments, if extensive adaptation continues in the FI20-G30 strain, changes could occur in the Mal31p transporter, potentially reaching the sequence of transporters grouped in set 1, similar to what might have occurred in the SpIB2 brewing strains and the strain containing ScMalt#5p.

## 4 Conclusions

On the one hand, our results strongly support adaptive selection as a powerful tool for obtaining strains with improved characteristics. On the other hand, we propose, based on our *in silico* analysis, that the residue at position 371 in maltose and maltotriose transporters is a key element distinct from those previously reported. Our bioinformatics predictions support the notion that alterations in TMH7 and TMH11 of these transporters play a very important role in the characteristics of the protein, which could, in turn, be reflected in changes in its transport activity [12,17]. These results emphasize the importance of focusing on specific polymorphisms in MFS transporter sequences, particularly in helices critical for substrate preference and specificity for industrial and biotechnological applications.

## Supplementary Material

Supplementary material
