# Supplementary material for: Computational analysis of polymorphic residues in maltose and maltotriose transporters of a wild Saccharomyces cerevisiae strain
Source: Open Life Sci. 2025 Apr 16;20(1):20251080. doi: 10.1515/biol-2025-1080 (PMC12032978; doi:10.1515/biol-2025-1080)
Supplement: Supplementary material [file biol-2025-1080-sm.pdf]

Supplementary material

Table S1: Primers used for PCR analysis

Name	Target	Sequence (5′-3′)	Amplicon size (b)	Source
ScMALx1	*MAL*x1	GGTATTAACAGGAGAAGAACGAG TGTCTGAACATCCTAAACCACC	282	(Magalhães et al., 2016).
ScAGT1	*AGT*1	GTCCATATTAGTGTCTACTACCCT	128	
		GTAATTTCGTAAGAACCCTCCC		
MPHx	*MPH*x	CGACAGCATTGAATACATATCCC ATATAACATAAGGTCAGCCCGA	204	
MTT1	*MTT*1	TTGTTTATGTTGGGTCGGTC	201	
		CAATCCAAATGCGTAAAGGTC		
ITS1	ITS-*5.8S*	TCCGTAGGTGAACCTGCGG	836–880	(White et al., 1990)
ITS4		TCCTCCGCTTATTGATATGC		

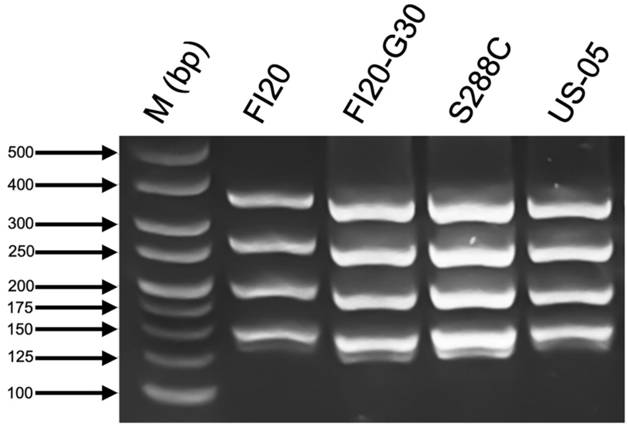

Figure S1: Restriction patterns of ITS-5.8S in FI20 and FI20-G30 strains using the *Hae*III enzyme. The restriction pattern sizes observed match those of *S. cerevisiae* (approximately 320, 240, 180, and 140 bp). Additionally, they show similarity to our control *S. cerevisiae* strains, S288C and US-05.

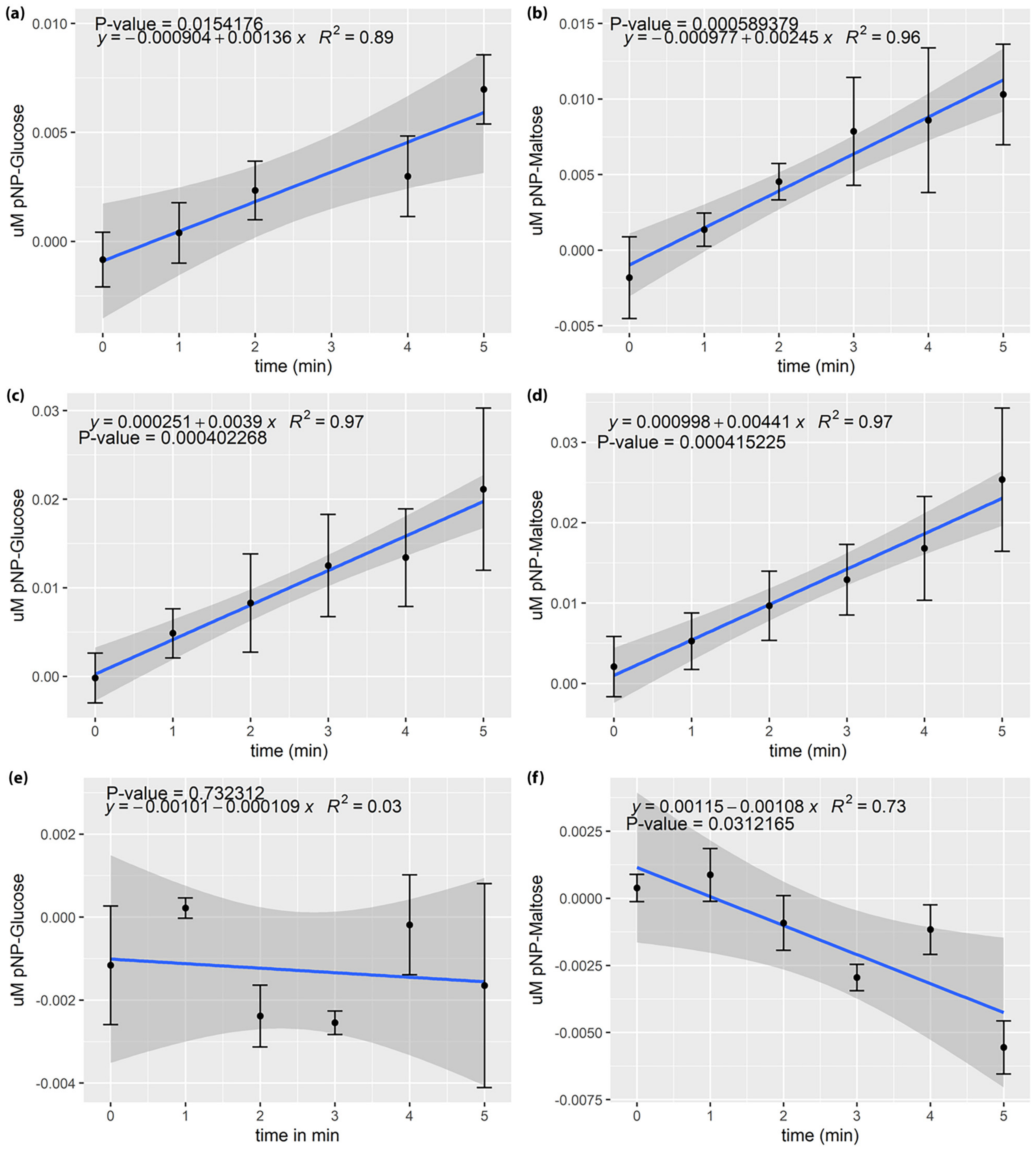

Figure S2: Cellular transport rate of pNP-Glucose and pNP-Maltose for 5 minutes. (a) and (b) correspond to the FI20 strain. (c) and (d) correspond to the FI20-G30 strain. (e) and (f) correspond to the control strain S288C, in which no transport was detected for either of the substrates used.

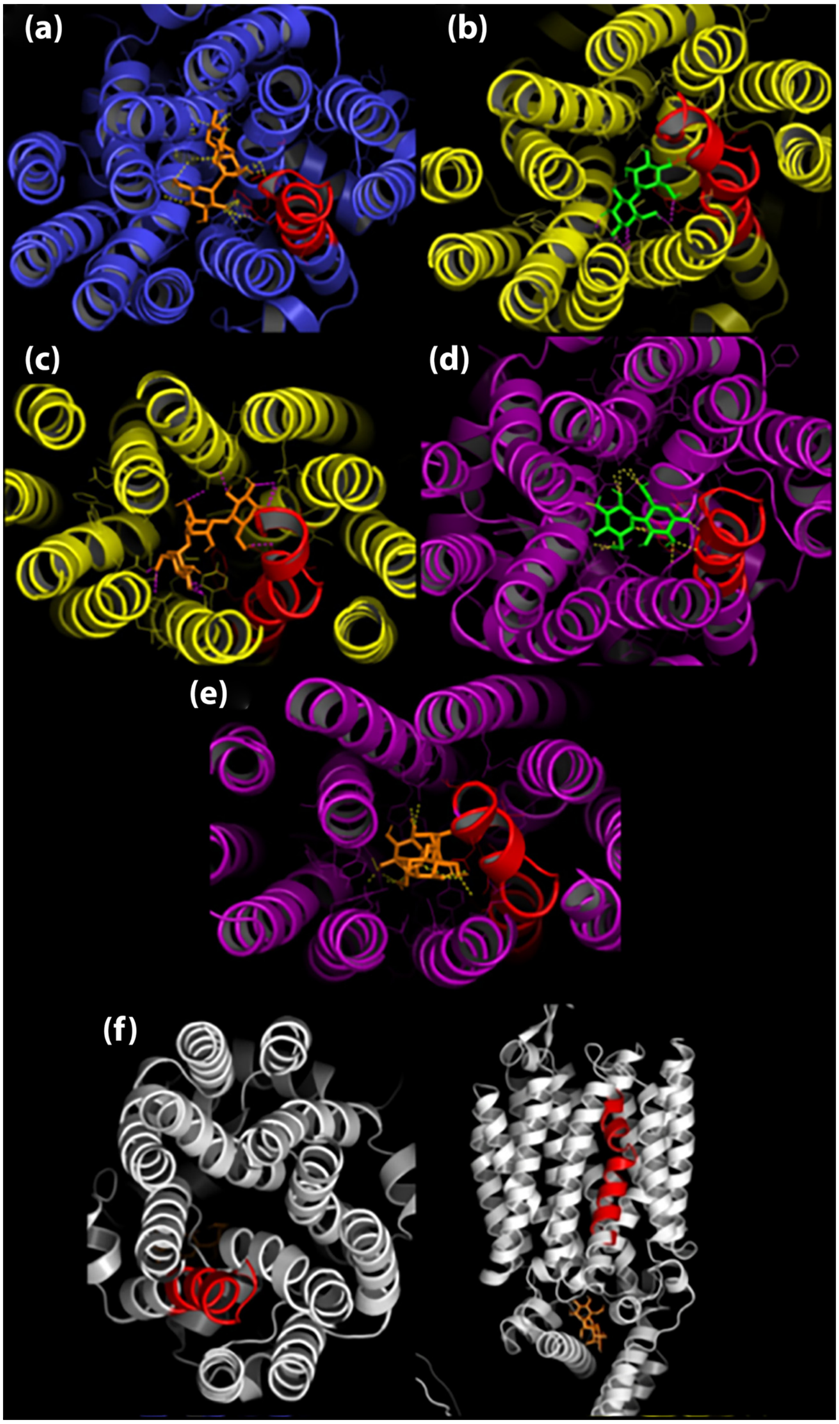

Figure S3: Molecular docking between transporters and maltose and maltotriose. (a) Mal31p-288 with maltotriose. (b) Mtt1p-1 with maltose. (c) Mtt1p-1 with maltotriose. (d) Mal31p-3-SpIB2 with maltose. (e) Mal31p-3-SpIB2 with maltotriose. (f) Mal31p-FI20/G30 with maltotriose. TMH7 is marked in red in all images.
